# Supplementary material for: Retinal structure and related factors in 8-year-old Japanese children: The Yamanashi adjunct study of the Japan Environment and Children’s Study
Source: PLoS One. 2025 May 12;20(5):e0323641. doi: 10.1371/journal.pone.0323641 (PMC12068617; doi:10.1371/journal.pone.0323641)
Supplement: S1 File — S2 Table. A quadrant-by-quadrant comparison for each layer. S3 Table. Average macular thickness. S4 Table. Thickness of the choroid. S5 Table. Thickness of ONL to BM. S6 Table. Thickness of GC-IPL. S7 Table. Thickness of NFL. S8 Figure. Scatter plots of Average macular thickness in all sectors versus AL and body height. a) AL vs. Fovea, b) AL vs. Average macular thickness whole sector, c) AL vs. Average macular thickness central sector, d) AL vs. Average macular thickness inner sector, e) AL vs. Average macular thickness outer sector, f) Body height vs. Fovea, g) Body height vs. Average macular thickness whole sector, h) Body height vs. Average macular thickness central sector, i) Body height vs. Average macular thickness inner sector, j) Body height vs. Average macular thickness outer sector. S9 Table: Multivariable regression analysis of the relationship between ONL to BM thickness and axial length, sex, and body height. S10 Table: Multivariable regression analysis of the relationship between GC-IPL thickness and axial length, sex, and body height. S11 Table: Multivariable regression analysis of the relationship between NFL thickness and axial length, sex, and body height. S12 Table: Comparison of left and right eye differences in average macular thickness. S13 Table: Comparison of left and right eye differences in choroidal thickness. S14 Table: Comparison of left and right eye differences in ONL to BM thickness. S15 Table: Comparison of left and right eye differences in GC-IPL thickness. S16 Table: Comparison of left and right eye differences in NFL thickness. (DOCX) [file pone.0323641.s001.docx]

**S1 Comparison of included and excluded groups.**

|  | Included  (n=304) | Excluded  (n=255) | P |
| --- | --- | --- | --- |
| AL (mm) | 23.06±0.74  (21.95-24.31) | 23.22±0.87  (21.84-24.62) | 0.01 ^a^ |
| SD (D) | -0.32±0.77  (-1.76-+0.63) | -0.92±1.91  (-4.36-+0.79) | 0.001 ^a^ |
| Uncorrected logMAR | 0.07±0.19  (0.00-0.52) | 0.14±0.28  (0.00-0.82) | 0.001 ^a^ |
| Body height (cm) | 125.2±5.1  (117.9-133.4) | 125.1±4.9  (117.1-132.8) | 0.82 ^a^ |
| Boy  Girl | 150  154 | 127  128 | 0.98 ^b^ |

Data are the mean ± standard deviation (5th–95th percentile).

^a^ P values are comparisons of included and excluded groups by independent samples t-test.

^b^ P values are comparisons of included and excluded groups by χ^2^ test.

.

**S2 A quadrant-by-quadrant comparison for each layer.**

|  | **Average macular thickness** | **ONL to BM** | **GC-IPL** | **NFL** |
| --- | --- | --- | --- | --- |
|  | P^a^ | P^a^ | P^a^ | P^a^ |
| Nasal vs. Inferior | < 2e-16 | 6.5e-10 | < 2e-16 | < 2e-16 |
| Superior vs. Inferior | 8.4e-15 | 0.03 | 0.36 | 0.001 |
| Superior vs. Nasal | < 2e-16 | 0.001 | < 2e-16 | < 2e-16 |
| Temporal vs. Inferior | 4.3e-12 | < 2e-16 | 1.00 | < 2e-16 |
| Temporal vs. Nasal | < 2e-16 | 5.2e-05 | < 2e-16 | < 2e-16 |
| Temporal vs. Superior | < 2e-16 | 3.2e-15 | 1.00 | < 2e-16 |

^a^ One way ANOVA.

**S3 Average macular thickness.**

|  | **All participants**  **(n=304)** | **Boy**  **(n=150)** | **Girl**  **(n=154)** |
| --- | --- | --- | --- |
| Thickness (㎛)  Whole sector | 309.7±10.9 (292-328) | 311.4±11.3 (293-331) | 308.1±10.3 (292-326) |
| Fovea | 220.9±15.3 (196-249) | 222.0±16.5 (196-250) | 219.8±14.0 (199-245) |
| Central sector | 257.9±15.8 (233-285) | 260.0±16.3 (234-287) | 255.9±15.1 (232-280) |
| Inner sector | 335.9±12.2 (319-356) | 338.1±12.4 (321-357) | 333.8±11.7 (316-354) |
| Outer sector | 296.5±11.6 (277-317) | 297.5±12.4 (277-318) | 295.6±10.7 (279-313) |
| Inner temporal | 326.6±12.2 (308-347) | 329.2±12.0 (311-350) | 324.1±11.8 (306-342) |
| Inner superior | 341.1±12.7 (322-363) | 342.8±13.1 (323-363) | 339.4±12.0 (322-359) |
| Inner nasal | 341.7±13.2 (321-363) | 343.5±13.4 (322-365) | 340.0±12.8 (321-360) |
| Inner inferior | 334.3±12.7 (316-357) | 337.1±13.0 (320-359) | 331.7±11.9 (313-352) |
| Outer temporal | 283.3±11.7 (264-302) | 285.9±11.8 (266-305) | 280.7±11.0 (263-299) |
| Outer superior | 298.0±12.3 (277-322) | 298.2±13.1 (276-323) | 297.8±11.5 (279-317) |
| Outer nasal | 315.5±13.4 (294-339) | 315.7±14.4 (294-340) | 315.2±12.4 (295-337) |
| Outer inferior | 289.3±12.2 (269-309) | 290.1±13.0 (269-310) | 288.5±11.4 (269-307) |

Data are the mean ± standard deviation (5th–95th percentile).

**S4 Thickness of the choroid.**

|  | **All participants**  **(n=239)** | **Boy(n=118)** | **Girl(n=121)** |
| --- | --- | --- | --- |
| Thickness (㎛)  Choroid | 301.5±34.0 (241-357) | 300.7±33.8 (237-357) | 302.3±34.3 (246-355) |

Data are the mean ± standard deviation (5th–95th percentile).

**S5 Thickness of ONL to BM.**

|  | **All participants**  **(n=304)** | **Boy(n=150)** | **Girl(n=154)** |
| --- | --- | --- | --- |
| Thickness (㎛)  Whole sector | 134.8±6.8 (124-145) | 135.6±7.0 (124-147) | 133.9±6.6 (123-145) |
| Central sector | 161.1±9.7 (146-177) | 162.0±10.2 (146-178) | 160.3±9.0 (145-175) |
| Inner sector | 134.9±7.5 (123-147) | 135.6±7.5 (123-148) | 134.3±7.4 (122-146) |
| Outer sector | 128.0±6.4 (117-139) | 129.0±6.6 (117-140) | 127.0±6.2 (117-137) |
| Inner temporal | 140.2±8.6 (125-155) | 141.2±8.6 (127-156) | 139.3±8.5 (123-152) |
| Inner superior | 130.1±10.1 (113-147) | 129.9±10.0 (113-147) | 130.2±10.2 (114-146) |
| Inner nasal | 135.8±11.2 (117-154) | 136.4±11.4 (118-155) | 135.2±10.9 (117-154) |
| Inner inferior | 133.6±9.5 (117-148) | 134.8±9.6 (118-149) | 132.4±9.4 (117-146) |
| Outer temporal | 130.6±6.9 (119-141) | 131.8±6.9 (120-142) | 129.3±6.6 (118-140) |
| Outer superior | 129.8±7.2 (119-141) | 130.4±7.0 (120-142) | 129.2±7.3 (118-141) |
| Outer nasal | 129.1±7.8 (117-142) | 129.9±8.0 (117-143) | 128.3±7.4 (118-140) |
| Outer inferior | 122.6±6.9 (111-133) | 124.0±7.2 (111-135) | 121.2±6.3 (111-130) |

Data are the mean ± standard deviation (5th–95th percentile).

**S6 Thickness of GC-IPL.**

|  | **All participants**  **(n=304)** | **Boy (n=150)** | **Girl (n=154)** |
| --- | --- | --- | --- |
| Thickness (㎛)  Whole sector | 76.1±4.5 (70-84) | 76.5±4.7 (69-84) | 75.8±4.2 (70-83) |
| Inner sector | 89.2±5.2 (82-98) | 89.8±5.4 (81-99) | 88.6±5.0 (82-97) |
| Outer sector | 63.1±4.4 (57-71) | 63.1±4.6 (56-72) | 63.0±4.2 (57-70) |
| Inner temporal | 85.1±5.8 (76-96) | 86.1±5.9 (77-98) | 84.1±5.5 (76-93) |
| Inner superior | 90.5±5.6 (82-99) | 91.1±5.8 (81-101) | 90.0±5.4 (82-98) |
| Inner nasal | 91.6±5.9 (83-101) | 92.0±6.1 (82-102) | 91.3±5.8 (83-101) |
| Inner inferior | 89.5±5.4 (82-99) | 90.1±5.6 (82-101) | 88.9±5.1 (82-96) |
| Outer temporal | 65.0±5.2 (57-74) | 65.6±5.2 (57-75) | 64.5±5.1 (57-72) |
| Outer superior | 60.2±4.9 (53-69) | 60.3±5.0 (52-69) | 60.1±4.7 (53-68) |
| Outer nasal | 67.2±5.1 (59-76) | 67.1±5.5 (58-77) | 67.4±4.7 (60-75) |
| Outer inferior | 59.8±5.0 (52-69) | 59.5±5.3 (51-68) | 60.1±4.8 (52-69) |

Data are the mean ± standard deviation (5th–95th percentile).

**S7 Thickness of NFL.**

|  | **All participants**  **(n=304)** | **Boy (n=150)** | **Girl (n=154)** |
| --- | --- | --- | --- |
| Thickness (㎛)  Whole sector | 29.1±2.1 (26-33) | 28.8±1.9 (26-32) | 29.4±2.2 (26-33) |
| Inner sector | 23.4±1.7 (21-26) | 23.5±1.7 (21-26) | 23.4±1.6 (21-26) |
| Outer sector | 34.8±3.3 (30-40) | 34.2±2.8 (29-38) | 35.4±3.6 (30-42) |
| Inner temporal | 18.8±2.4 (15-23) | 18.8±2.4 (15-23) | 18.8±2.4 (15-22) |
| Inner superior | 27.1±2.2 (23-31) | 26.9±2.1 (23-30) | 27.2±2.3 (24-31) |
| Inner nasal | 22.6±2.3 (19-26) | 22.6±2.2 (19-26) | 22.7±2.4 (19-26) |
| Inner inferior | 25.2±2.7 (21-29) | 25.6±2.8 (21-31) | 24.9±2.6 (21-29) |
| Outer temporal | 18.6±2.7 (14-23) | 18.7±2.8 (14-23) | 18.5±2.6 (14-22) |
| Outer superior | 37.4±4.5 (31-46) | 36.3±3.4 (31-42) | 38.5±5.1 (32-47) |
| Outer nasal | 45.7±4.9 (38-54) | 44.9±4.2 (38-52) | 46.5±5.4 (39-55) |
| Outer inferior | 37.6±4.2 (31-45) | 37.0±3.8 (31-43) | 38.2±4.5 (32-46) |

Data are the mean ± standard deviation (5th–95th percentile).

**S9 Multivariable regression analysis of the relationship between ONL to BM thickness and axial length, sex, and body height.**

|  | **AL** | | | | | **Sex** | | | | | **Body height** | | | | |
| --- | --- | --- | --- | --- | --- | --- | --- | --- | --- | --- | --- | --- | --- | --- | --- |
| **Thickness** | **B ^a^** | **95%CI of B** | | **β ^b^** | **P** | **B ^a^** | **95%CI of B** | | **β ^b^** | **P** | **B ^a^** | **95%CI of B** | | **β ^b^** | **P** |
| Whole | -1.24 | -2.34 | -0.13 | -0.13 | 0.02 | -2.30 | -3.91 | -0.68 | -0.17 | 0.005 | 0.03 | -0.12 | 0.19 | 0.03 | 0.66 |
| Central | -0.37 | -1.95 | 1.22 | -0.03 | 0.64 | -1.94 | -4.24 | 0.37 | -0.10 | 0.09 | 0.17 | -0.05 | 0.39 | 0.09 | 0.12 |
| Inner | -1.51 | -2.73 | -0.30 | -0.15 | 0.01 | -2.08 | -3.85 | -0.31 | -0.14 | 0.02 | 0.03 | -0.14 | 0.20 | 0.02 | 0.70 |
| outer | -1.18 | -2.22 | -0.14 | -0.14 | 0.02 | -2.61 | -4.13 | -1.09 | -0.20 | <0.001 | <0.001 | -0.14 | 0.14 | <0.001 | 0.99 |

Sex was analyzed by assigning boys a value of 0 and girls a value of 1.

^a^ Nonstandardized Regression Coefficient B.

^b^ Standardized Regression Coefficient β.

**S10 Multivariable regression analysis of the relationship between GC-IPL thickness and axial length, sex, and body height.**

|  | **AL** | | | | | **Sex** | | | | | **Body height** | | | | |
| --- | --- | --- | --- | --- | --- | --- | --- | --- | --- | --- | --- | --- | --- | --- | --- |
| **Thickness** | **B ^a^** | **95%CI of B** | | **β ^b^** | **P** | **B ^a^** | **95%CI of B** | | **β ^b^** | **P** | **B ^a^** | **95%CI of B** | | **β ^b^** | **P** |
| Whole | -0.97 | -1.69 | -0.24 | -0.16 | 0.009 | -1.19 | -2.25 | -0.13 | -0.13 | 0.02 | 0.10 | 0.003 | 0.20 | 0.12 | 0.04 |
| Inner | -1.47 | -2.31 | -0.64 | -0.21 | <0.001 | -2.01 | -3.23 | -0.79 | -0.19 | 0.001 | 0.11 | -0.009 | 0.22 | 0.10 | 0.07 |
| outer | -0.45 | -1.17 | 0.28 | -0.07 | 0.22 | -0.36 | -1.42 | 0.70 | -0.04 | 0.50 | 0.10 | 0.003 | 0.20 | 0.12 | 0.04 |

Sex was analyzed by assigning boys a value of 0 and girls a value of 1.

^a^ Nonstandardized Regression Coefficient B.

^b^ Standardized Regression Coefficient β.

**S11 Multivariable regression analysis of the relationship between NFL thickness and axial length, sex, and body height.**

|  | **AL** | | | | | **Sex** | | | | | **Body height** | | | | |
| --- | --- | --- | --- | --- | --- | --- | --- | --- | --- | --- | --- | --- | --- | --- | --- |
| **Thickness** | **B ^a^** | **95%CI of B** | | **β ^b^** | **P** | **B ^a^** | **95%CI of B** | | **β ^b^** | **P** | **B ^a^** | **95%CI of B** | | **β ^b^** | **P** |
| Whole | -0.14 | -0.48 | 0.20 | -0.05 | 0.42 | 0.50 | <0.001 | 0.99 | 0.12 | 0.05 | 0.03 | -0.01 | 0.08 | 0.08 | 0.15 |
| Inner | 0.06 | -0.21 | 0.34 | 0.03 | 0.65 | -0.02 | -0.41 | 0.38 | -0.004 | 0.93 | 0.002 | -0.04 | 0.04 | 0.008 | 0.88 |
| outer | -0.34 | -0.87 | 0.19 | -0.08 | 0.20 | 1.00 | 0.23 | 1.78 | 0.15 | 0.01 | 0.07 | -0.007 | 0.14 | 0.10 | 0.07 |

Sex was analyzed by assigning boys a value of 0 and girls a value of 1.

^a^ Nonstandardized Regression Coefficient B.

^b^ Standardized Regression Coefficient β.

**S12 Comparison of left and right eye differences in average macular thickness.**

|  | **Right eye**  **(n=186)** | **Left eye**  **(n=118)** | **P ^a^** |
| --- | --- | --- | --- |
| Thickness (㎛)  Whole sector | 309.8 ±10.6 (292-326) | 309.6±11.5 (294-331) | 0.651 |
| Fovea | 221.0 ±15.9 (196-245) | 220.7±14.3 (200-250) | 0.847 |
| Central sector | 257.8 ±16.7 (232-285) | 258.1±14.3 (238-284) | 0.974 |
| Inner sector | 336.1 ±11.8 (319-355) | 335.6±12.9 (319-357) | 0.578 |
| Outer sector | 296.6±11.1 (277-316) | 296.4±12.4 (276-318) | 0.685 |
| Inner temporal | 326.7±12.0 (308-346) | 326.3±12.5 (309-348) | 0.639 |
| Inner superior | 340.9±12.1 (322-360) | 341.5±13.6 (323-363) | 0.935 |
| Inner nasal | 342.2±12.8 (322-361) | 340.8±13.9 (321-365) | 0.266 |
| Inner inferior | 334.6±12.3 (317-354) | 333.9±13.3 (316-359) | 0.463 |
| Outer temporal | 283.9±11.5 (265-303) | 282.3±12.0 (263-301) | 0.251 |
| Outer superior | 297.9±11.7 (278-316) | 298.2±13.3 (276-323) | 0.915 |
| Outer nasal | 315.0±12.5 (295-337) | 316.2±14.7 (293-340) | 0.721 |
| Outer inferior | 289.6±12.1 (269-310) | 288.8±12.5 (269-308) | 0.511 |

Data are the mean ± standard deviation (5th–95th percentile).

^a^ Mann-Whitney U test.

|  | **Right eye**  **(n=144)** | **Left eye**  **(n=95)** | **P ^a^** |
| --- | --- | --- | --- |
| Thickness (㎛)  Choroid | 303.4±36.3 (238-362) | 298.6±30.1 (247-347) | 0.273 |

**S13 Comparison of left and right eye differences in choroidal thickness.**

Data are the mean ± standard deviation (5th–95th percentile).

^a^ Mann-Whitney U test.

**S14 Comparison of left and right eye differences in ONL to BM thickness.**

|  | **Right eye**  **(n=186)** | **Left eye**  **(n=118)** | **P ^a^** |
| --- | --- | --- | --- |
| Thickness (㎛)  Whole sector | 134.7±6.7 (123-145) | 134.8±7.0 (125-147) | 0.947 |
| Central sector | 161.0±9.2 (147-176) | 161.3±10.4 (143-178) | 0.866 |
| Inner sector | 134.8±7.3 (122-146) | 135.1±7.7 (124-148) | 0.875 |
| Outer sector | 128.1±6.4 (117-138) | 127.9±6.6 (118-140) | 0.824 |
| Inner temporal | 140.8±8.7 (125-155) | 139.4±8.4 (124-152) | 0.117 |
| Inner superior | 129.9±9.9 (113-145) | 130.3±10.5 (115-148) | 0.974 |
| Inner nasal | 134.9±11.2 (117-154) | 137.2±11.0 (119-156) | 0.079 |
| Inner inferior | 133.7±9.8 (116-149) | 133.5±9.1 (118-147) | 0.830 |
| Outer temporal | 130.9±6.9 (118-142) | 130.0±6.8 (119-141) | 0.181 |
| Outer superior | 129.7±7.1 (119-141) | 129.9±7.4 (120-142) | 0.879 |
| Outer nasal | 128.8±7.7 (117-140) | 129.5±7.8 (118-142) | 0.516 |
| Outer inferior | 122.8±6.9 (111-134) | 122.2±6.9 (111-132) | 0.448 |

Data are the mean ± standard deviation (5th–95th percentile).

^a^ Mann-Whitney U test.

**S15 Comparison of left and right eye differences in GC-IPL thickness.**

|  | **Right eye**  **(n=186)** | **Left eye**  **(n=118)** | **P ^a^** |
| --- | --- | --- | --- |
| Thickness (㎛)  Whole sector | 75.9±4.5 (69-84) | 76.5±4.4 (70-84) | 0.260 |
| Inner sector | 88.9±5.2 (82-98) | 89.7±5.3 (82-98) | 0.205 |
| Outer sector | 62.9±4.5 (57-70) | 63.3±4.3 (57-72) | 0.364 |
| Inner temporal | 84.6±5.7 (76-96) | 85.8±5.7 (77-96) | 0.071 |
| Inner superior | 90.2±5.5 (82-99) | 91.0±5.7 (82-99) | 0.184 |
| Inner nasal | 91.6±6.0 (83-102) | 91.7±5.9 (83-101) | 0.974 |
| Inner inferior | 89.1±5.3 (81-99) | 90.1±5.6 (82-99) | 0.141 |
| Outer temporal | 65.1±5.3 (57-74) | 65.0±5.1 (56-73) | 0.888 |
| Outer superior | 60.0±4.8 (53-68) | 60.5±4.9 (53-69) | 0.332 |
| Outer nasal | 66.9±5.0 (59-75) | 67.8±5.2 (58-78) | 0.150 |
| Outer inferior | 59.8±5.2 (51-69) | 59.9±4.8 (53-68) | 0.611 |

Data are the mean ± standard deviation (5th–95th percentile).

^a^ Mann-Whitney U test.

**S16 Comparison of left and right eye differences in NFL thickness.**

|  | **Right eye**  **(n=186)** | **Left eye**  **(n=118)** | **P ^a^** |
| --- | --- | --- | --- |
| Thickness (㎛)  Whole sector | 29.2±2.1 (26-33) | 29.0±2.1 (26-33) | 0.540 |
| Inner sector | 23.5±1.6 (21-26) | 23.3±1.7 (20-26) | 0.171 |
| Outer sector | 34.8±3.3 (30-40) | 34.8±3.3 (29-40) | 0.963 |
| Inner temporal | 18.8±2.3 (15-23) | 18.7±2.6 (15-23) | 0.918 |
| Inner superior | 27.2±2.2 (24-31) | 26.8±2.3 (23-30) | 0.316 |
| Inner nasal | 22.9±2.2 (19-26) | 22.3±2.4 (19-26) | 0.039 |
| Inner inferior | 25.2±2.6 (21-29) | 25.2±2.9 (21-30) | 0.755 |
| Outer temporal | 18.7±2.6 (14-23) | 18.5±2.8 (14-22) | 0.844 |
| Outer superior | 37.6±4.6 (32-46) | 37.0±4.2 (31-44) | 0.571 |
| Outer nasal | 45.6±4.9 (38-54) | 45.8±5.0 (38-53) | 0.742 |
| Outer inferior | 37.5±4.2 (31-44) | 37.8±4.2 (32-45) | 0.600 |

Data are the mean ± standard deviation (5th–95th percentile).

^a^ Mann-Whitney U test.
